# Supplementary material for: Effect of safranal or candesartan on 3-nitropropionicacid-induced biochemical, behavioral and histological alterations in a rat model of Huntington’s disease
Source: PLoS One. 2023 Nov 1;18(11):e0293660. doi: 10.1371/journal.pone.0293660 (PMC10619823; doi:10.1371/journal.pone.0293660)
Supplement: Attachment — Submitted filename: PONE-D-23-06048.docx [file pone.0293660.s001.docx]

**REVIEW REPORT**

**Manuscript number:** PONE-D-23-06048

**Full Title:** Effect of safranal or candesartan on 3-nitropropionicacid-induced biochemical, behavioral and histological alterations in a rat model of Huntington’s disease

Authors assessed the effect of safranal and candesartan on 3-nitropropionicacid-induced biochemical, behavioral and histological alterations in a rat model of Huntington’s disease. This is an interesting subject area. Based on the findings of the research, authors concluded that safranal and candesartan may prevent or delay the progression of HD and its associated impairments through their antioxidant, anti-inflammatory, anti-apoptotic and neuromodulator effects. Although the manuscript has been well written, some clarifications or inputs need to be made in order to greatly improve the quality of this manuscript. Below are some of the comments.

**Abstract**

1. A statement on the potential neuroprotective effects of safranal and candesartan should be inserted before the aim to justify why these drugs were chosen.
2. The doses of the test compounds should be well written. Are authors referring to 50 mg/kg for safranal and 1 mg/kg for candesartan? In line 48, authors should indicate the route of administration for 3-NP.
3. The sentence in Lines 48-50 is quite confusing. Description of methods should always be precise and easy to apprehend. Authors should kindly revise.

**Introduction**

4. In line 65, CAG should be defined. It is always important to define abbreviations in a text on first mention. The sentence captured in lines 88-90 is incomplete. Please revise.

**Materials and Methods**

5. In addition to weight, can authors state the age of animals. Were the animals housed individually or in groups during the acclimatization period? If in groups, how many were housed in a group? Also provide cage dimensions. Authors should indicate the start times of the light and dark cycle.
6. Number of animals (40) in a group are too many, bringing about issues regarding ethics. Why 40? How were drug solutions prepared and in what volume were they administered? What was the rationale of administering paraffin oil and CMC to some groups of rats? Why wasn’t a naïve control group introduced. How were drugs administered orally? What informed the choice of doses for safranal and candesartan? In addition, why did authors decide to use single doses for the test compounds? Why did authors choose to administer test compounds for 9 days?
7. Why did group 5 receive 3-NP from day 3 and not day 1? The statement “The sixth group (Safr+3-NP) received IP injection of 50 mg Safr/kg/day for 9 days followed by IP injection of 20 mg 3-NP/kg/day from the 3rd day to the 9th day’ is difficult to appreciate. Same can be said for group 7. Authors should clarify exactly how the dosing of 3-NP plus test compounds was done.
8. With regards to the behavioural tests performed, can authors indicate the exact start day and time of the experiments. For example, EPM was conducted on day 10 or 11 and at 9 a.m. after start of treatment etc.
9. OFT. Why was wood and not Plexiglas used for the OF apparatus? Authors indicate that the OFT was conducted in morning daylight. What was the exact time? Dimension for the OF apparatus: (90 x 90 x 25 cm^3^) or (90 cm x 90 cm x 90 cm) not (90 x 90 x 25 cm). same applies to the squares. What action was taken after the 5-minute session if an animal defecated in the arena? How were the parameters measured? The OFT is a conventional approach/avoidance paradigm in which anxiety and exploration are simultaneously evoked by a novel setting. An increase in activity or time spent in the center of the open field indicates reduction in anxiety and/or increase in exploration. It would have therefore been very important for authors to measure central activity.
10. EPM. The description indicates that anxiety behaviour was measured, not cognitive function. To assess cognitive function, the EPM transfer latency can be used. Please refer to literature. Was the apparatus made of wood or Plexiglas? How were the parameters recorded? With regards to dimensions, refer to comments on OF.
11. Experiments to measure movement behaviour should have been performed to assess the effects of safranal and Candesartan on motor abnormalities since this is an important trait of HD. Refer to Sidhu et al, 2018.
12. With regards to the one-way ANOVA, which post hoc test was used for multiple comparisons.

**Results**

13. What were the observed effects after 3-NP administration?
14. In line 277, which corresponding control group are authors referring to?
15. Statistical analysis e.g. p values are missing in the description.
16. Too many tables. Presentation of results as a blend of figures and tables would have been the best. Figures most often are easy to interpret. Ill suggest that some results from the behavioural tests are presented as figures.
17. For all tables, the statement “Data is displayed as mean ± standard error (SE)” can be captured in the legend.
18. For all tables, authors should note that the significant differences that exist with regards to the various comparisons featured in the legend have not been duly captured. For instance, **e:** represents significant difference as compared to 3-NP-treated group. Looking, at the values for PO or CMC in table 2, there should be significance in some of the parameters.
19. With regards to figs 1-4 (Photomicrograph of the striatum for the various treatments), authors can show in the micrographs, the normal and histological changes of neurons in the striatum for better appreciation.

**Discussion**

20. Authors made reference to a study by Fotoohi et al, regarding the beneficial effects of Safranal on movement defects induced by 3-NP during the two weeks of experiment. The doses used in this study are quite higher that that study (0.75, 1.5 and 3.0 mg/kg)? Why didn’t authors use the same or similar doses since beneficial effects were achieved with them.
21. There is silence on the relevance of assessing the effects of the test compounds in the EPM and OFT. A detailed discussion is needed. As stated earlier, EPM test measures anxiety behaviour but can also be used to measure cognitive function in the transfer latency test. This wasn’t done. It is therefore surprising authors are relating EPM to cognitive impairment.
22. In paragraph 2, authors conclude by stating “the current results showed that 3-NP injection significantly upregulated the mRNA levels of MAO-A and –B.” What about the effects of test compounds? Authors should focus their discussion on the benefits of test drugs based on the results.
