# Supplementary material for: Effect of safranal or candesartan on 3-nitropropionicacid-induced biochemical, behavioral and histological alterations in a rat model of Huntington’s disease
Source: PLoS One. 2023 Nov 1;18(11):e0293660. doi: 10.1371/journal.pone.0293660 (PMC10619823; doi:10.1371/journal.pone.0293660)
Supplement: Attachment — Submitted filename: Response to reviewers R2 222.docx [file pone.0293660.s003.docx]

**Ref.:**  Ms. No. **PONE-D-23-06048R1**

**Title:** **“Effect of safranal or candesartan on 3-nitropropionicacid-induced biochemical, behavioral and histological alterations in a rat model of Huntington’s disease”.**

**Journal:** PLOS ONE

*Dear editor-in chief*,

*Respected Editor of* *The Journal of* PLOS ONE

We appreciate the valuable inputs given by the respected reviewers which helped us greatly to improve the manuscript. We have tackled all the recommended changes. Below, you will find answers item-by-item to the reviewers’ comments:

**Dear respected reviewers,**

On behalf of my coauthors, I am so grateful to you for your thorough revision and helpful insights that will certainly support our manuscript.

**Reviewer #2:**

**Introduction**

**Comment #1:** The sentence captured in lines 91-93 ‘’Ang II type 1 receptor (AT1R) blockers (ARBs) have been reported to be beneficial in attenuating cognitive deficits observed in Alzheimer’s disease, Parkinson’s disease, vascular’’ is incomplete. Please revise.

**Response:** In the revised manuscript, **Introduction** section, page 3, lines 92-94, the captured sentence was revised as follows:

"Ang II type 1 receptor (AT1R) blockers (ARBs) have been reported to be useful in diminishing cognitive deficits linked to Post-Stroke Cognitive Impairment (PSCI), Alzheimer’s Disease, Parkinson’s Disease, and Vascular Cognitive Impairment (VCI)"

**Materials and Methods**

**Comment #2:** Details regarding preparation of the various drug solutions should be outlined in the manuscript (This is captured in your response)**.**

**Response:** In the revised manuscript, **M&M** section, page 4, lines 114-121, the details of drug preparations were stated for each of the applied drugs.

**Comment #3:** For the experimental design/procedure, authors can represent the various treatments as Safr 50 mg/kg/day rather than 50 mg Safr/kg/day.

**Response:** In the revised manuscript, **M&M** section, page 5, lines 134-140, the rcommended comments in the experimental design/procedure were considered.

**Comment #4:** For the various dimensions (cm3), 3 should be in superscript.

**Response:** In the revised manuscript, **M&M** section, page 4 (line 125), page 6 (lines 154- 171), action was taken as per comment.

**Comment #5:** With regards to the behavioural testing, authors indicate that the training session for the passive avoidance test started at 6:00 pm on training day 9 in which a 0.5 mA with 50 Hz foot electric shock was delivered for two seconds. The behavioural assessment in the EPM & OFT was however done on day 10 of treatment. Couldn’t this have influenced the results seen with regards to the EPM and OFT? In line 149, can authors state the % alcohol used?

**Response:** in the revised manuscript, **M&M** section (fig 1), pages 5, line 139, in the summary of the experimental design, a flowchart was added to show that each of the behavioural tests (OFT, EPM & PA) was conducted using different set of the experimental animals (n=7, per group, per test).

Actually, we had two options; option #1 is to conduct the OFT and EPM on the 10^th^ day, then to run the training session of PA, on the 11^th^ day, then the main experiment of PA on the 12^th^ day. Accordingly, three days would be passed after the last treatment. thus, the results of PA may not be representative for the conducted treatment.

Option #2 is to conduct the behavioural experiments using different set of the experimental animals (n=7, per group, per test), to avoid the effect of the electrical shock in the PA on the results of the other behavioral tests. We chosen the second option, because it gave us the opportunity to apply all the beahvioural tests on the 10^th^ day and to avoid that any of the studied behaviors may be affected by the other behavioural tests.

In the revised manuscript, M&M section, page 6, line 161, the % of alcohol was stated as 70%.

**Comment #6:** In Fig 1, the experimental design should capture a detailed sequential summary of all experiments from start to finish. This includes treatments, behavioural assessments & sample analysis.

**Response**: in the revised manuscript, **M&M** section, page 5, lines 139-140, the detailed sequential summary of all the experiments was stated as per comment.

**Results**

**Comment #7:** What were the observed effects after 3-NP administration?

**Response:** in the revised manuscript, **Results** section, page 10, lines 277-280, a brief description of the the effect of 3-NP injection was written. In addition, in the subsequent paragarphs, the detailed description of the effect of 3-NP inection was written and highlighted in yellow.

**Comment #8:** Capital letters are used to represent the outcomes of the statistical analysis. Can authors use symbols instead? Maybe *, #, etc.

**Response:** As clarified in page 10, line 267, under M&M-statistical analysis, the applied post-hoc test was Duncan's test to study the statistical homogeneity among all the experimental groups. Accordingly, letters are best fitting to represent the outcomes. However, to use symbols like "*, #,…", are best fitting for the outcome of other statistical post-hoc tests like for example tukey HSD and LSD. Duncan's test gives us the opportunity to compare among all groups, using Alphabet letters (in ascending or desecending order), especially that the groups have multiple controls. However, the outcomes of LSD and HSD may cause some confusion because there will be at least 6 symbols (as compared to the PO-group, the CMC-group, the Safr-alone group, the Cands-alone group, 3NP-group and Safr+3NP group)

**Comment #9:** Symbol for seconds is “s”.

**Response:** In the revised manuscript, **M&M** section, page 6-7 (lines 157, 174-176), as well as in **Results** section, page 12 (Fig 3), page 14 (Fig 4) and page 15 (Fig 5), action was taken as per comment.

**Comment #10:** The y-axis of the various figures can be numbered without decimals.

**Response:** In the revised manuscript, **Results** section, page 11 (Fig 2), page 12 (Fig 3), page 14 (Fig 4) and page 15 (Fig 5), all decimals were removed as per comment.

**Comment #11:** Authors used different in-text citations for the figures. Eg. fig 2, fig(5), or Fig. 5. Authors should refer to the journal’s guidelines and be consistent.

**Response:** In the revised manuscript, pages 4-5 (Fig 1), under **M&M** section as well as in page 10-15 (Figs 2-5), under the **results** section, all Figs were adjusted according to the journal guidelines.

**Discussion**

**Comment #12:** There is silence on the relevance of assessing the effects of the test compounds in the EPM and OFT.

**Response:** In the revised manuscript, **in Discussion** section, page 26 (lines 565-576) and page 27 (lines 590-602), action was taken as per comment.

We hope that we have answered all the comments raised by the respected referees and followed their recommendations and adopted the manuscript on journal's guidelines/style. We think that our manuscript has been considerably improved because of these thoroughly revisions. We hope that our revised manuscript “**Effect of safranal or candesartan on 3-nitropropionicacid-induced biochemical, behavioral and histological alterations in a rat model of Huntington’s disease**” is now suitable for publication in your respected journal *“***PLOS ONE***”.*

We should like to take this opportunity to thank the editor for his dedication and support. We look forward to hearing from you regarding our submission.

Thank you for understanding and support

Respectfully

***Sherine Maher Rizk, PhD***

Biochemistry Department

Faculty of Pharmacy

Cairo University

E-mail: sherine.abdelaziz@cu.edu.eg
